# Real-time multi-view deconvolution

**DOI:** 10.1093/bioinformatics/btv387

**Published:** 2015-06-25

**Authors:** Benjamin Schmid, Jan Huisken

**Affiliations:** Max Planck Institute of Molecular Cell Biology and Genetics, 01307 Dresden, Germany

## Abstract

**Summary:** In light-sheet microscopy, overall image content and resolution are improved by acquiring and fusing multiple views of the sample from different directions. State-of-the-art multi-view (MV) deconvolution simultaneously fuses and deconvolves the images in 3D, but processing takes a multiple of the acquisition time and constitutes the bottleneck in the imaging pipeline. Here, we show that MV deconvolution in 3D can finally be achieved in real-time by processing cross-sectional planes individually on the massively parallel architecture of a graphics processing unit (GPU). Our approximation is valid in the typical case where the rotation axis lies in the imaging plane.

**Availability and implementation:** Source code and binaries are available on github (https://github.com/bene51/), native code under the repository ‘gpu_deconvolution’, Java wrappers implementing Fiji plugins under ‘SPIM_Reconstruction_Cuda’.

**Contact:** bschmid@mpi-cbg.de or huisken@mpi-cbg.de

**Supplementary information:** Supplementary data are available at *Bioinformatics* online.

## 1 Introduction

MV imaging is particularly useful in light-sheet microscopy where consecutive views are acquired in short succession, allowing reconstruction of entire developing organisms without artifacts (Huisken *et* *al.*, 2004). Due to the low photo-toxicity in light sheet microscopy, time-lapse experiments are oftentimes run over days and terabytes of data accumulate quickly. MV fusion is therefore particularly desirable to be performed in real-time to eliminate redundant information from different views. Best fusion results, however, are achieved by combining fusion with 3D deconvolution (Swoger *et* *al.*, 2007; Verveer *et* *al.*, 2007; Wu *et* *al.*, 2013). Although efficient Bayesian MV deconvolution based on the Richardson–Lucy (RL) algorithm has been shown recently to outperform existing methods in terms of fusion quality and convergence speed, it is still too slow for real-time processing of typical data volumes (Preibisch *et* *al.*, 2014).

The RL deconvolution iterations consist only of convolutions and pixel-wise arithmetic operations and could therefore be significantly accelerated using dedicated hardware such as a graphics processing unit (GPU). The large memory requirements of MV deconvolution, however, exceed the limited resources of modern GPUs even for moderate data sizes (Supplementary Note S1). Previous attempts therefore required splitting the data into blocks of appropriate size. Each block then either had to be transferred to and from the GPU in each RL iteration (Preibisch *et* *al.*, 2014), or blocks needed to share a considerable amount of overlap to avoid border artifacts (Temerinac-Ott *et* *al.*, 2011). Therefore, GPU-based implementations only achieved a three-times performance gain (Preibisch *et* *al.*, 2014).

## 2 Results

**Fig. 1.**
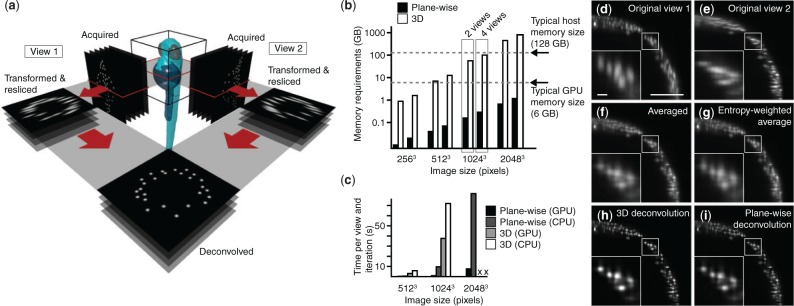
Plane-wise multi-view deconvolution concept and performance. (**a**) Concept of plane-wise deconvolution for two views. Each dataset is resliced into planes orthogonal to the microscope’s rotation axis. Datasets are deconvolved plane-by-plane. (**b**) Memory requirements for traditional 3D and our plane-wise multi-view deconvolution, for various data sizes and numbers of views, on a logarithmic scale. (**c**) Execution times for plane-wise multi-view deconvolution, implemented on GPU and CPU, and 3D deconvolution, with and without GPU support. Memory requirements for 3D deconvolution timings for the 2048^3^ pixel dataset were beyond the capabilities of our workstation. (**d–i**) Resulting images of a 9 h post-fertilization transgenic *Tg(h2afva:h2afva-mCherry)* zebrafish embryo, using different methods (view along the rotational axis, scale bar 100 µm, 10 µm in the inset): (**d, e**) acquired raw data, (**f–i**) fusion performed by (**f**) averaging, (**g**) entropy-weighted averaging, (**h**) 3D multi-view deconvolution and (**i**) plane-wise multi-view deconvolution (10 iterations). (Dell T6100, Intel E5-2630 @2.3 GHz 2 processors, 64 GB RAM; Graphics card: Nvidia GeForce GTX TITAN Black)

The primary goal of MV fusion is the improvement of the poor axial resolution in a single 3D dataset using the superior lateral resolution of an additional, overlapping dataset, and not necessarily to improve resolution beyond the intrinsic lateral resolution. We therefore approximated the full 3D point spread function (PSF) with a 2D PSF, neglecting one lateral component (along the rotation axis), and processed each plane orthogonal to the rotation axis independently (Fig. 1a). Memory requirements were thereby reduced by the number of lines read out from the camera chip, i.e. typically 100–1000 fold (Fig. 1b). This allowed us to implement the entire MV deconvolution on a GPU. Taking advantage of three CUDA (Compute Unified Device Architecture) streams, we interleaved GPU computations with data transfers, such that not only expensive copying to and from GPU memory, but also reading and writing data from and to the hard drive came without additional cost (Supplementary Note S2). Compared with 3D MV deconvolution, with and without GPU support, we thereby reduced processing times by a factor of up to 25 and 75, respectively (Fig. 1c, Supplementary Table S1), while producing comparable results.

We compared the results of our implementation to the methods commonly used in the light-sheet community, such as established 3D deconvolution (Preibisch *et* *al.*, 2014), averaging and entropy-based fusion (Preibisch *et* *al.*, 2010) (Fig. 1d–i). Both averaging and entropy-based fusion were blurry and showed cross-shaped artifacts, originating from the elongated PSFs along the optical axes. Three dimensional deconvolution and our plane-wise variant reduced artifacts and enhanced the contrast, thus truly improving the resolution in the fused dataset (Fig. 1h and i; Supplementary Fig. S1).

Although registration of the different views is still required, it can be performed in pre-processing before starting a time-lapse experiment, due to the repeatability of high-quality microscope stages. Multi-view deconvolution can then be performed in real time directly as the data is transferred from the camera.

We provide our software as a C library that can be directly linked to camera acquisition software for real-time processing, and as plugins for Fiji (Schindelin *et* *al.*, 2012) (Supplementary Material).

## 3 Validation

Our plane-wise deconvolution approximates 3D deconvolution by neglecting the contribution of the PSF along the rotation axis. It is therefore suited for systems with a single rotation axis lying within the imaging plane. Using artificial data (Supplementary Fig. S2 and Table S2), we confirmed the applicability of our approximation even if the rotation axis is slightly tilted (Supplementary Fig. S3). Its validity is independent of the amount of noise (Supplementary Fig. S4), but depends on the lateral extents of the PSF. Keeping its axial standard deviation fixed at eight pixels, a typical value measured on our microscopes, we found that up to a lateral standard deviation of 2–3 pixels, results from plane-wise and 3D deconvolution are undistinguishable (Supplementary Fig. S5). The measured lateral standard deviation of the PSF was typically between 1.5 and 1.8 pixels on our microscopes.

## 4 Conclusion

The photo-efficiency of light-sheet microscopy enables long time-lapse imaging of living samples to study fundamental questions in developmental biology. However, its huge data rates also open new challenges for data processing. A key problem in light-sheet microscopy has been the fusion of data recorded from multiple angles. In this article, we presented a new method that performs MV deconvolution plane-wise, which reduces memory requirements compared with existing methods and thus permits an entirely GPU-based implementation. The achieved acceleration makes MV deconvolution for the first time applicable in real-time without the need for data cropping or resampling.

## Supplementary Material

Supplementary Data
