# Supplementary material for: Real-time multi-view deconvolution
Source: Bioinformatics. 2015 Jun 25;31(20):3398–400. doi: 10.1093/bioinformatics/btv387 (PMC4595906; doi:10.1093/bioinformatics/btv387)
Supplement: Supplementary Data [file supp_31_20_3398__index.html]

Real-time multi-view deconvolution — Real-time multi-view deconvolution — Real-time multi-view deconvolution — Supplementary Data 

# Real-time multi-view deconvolution

## Supplementary Data

files

- Supplementary Data - pdf file
