# Supplementary material for: Real-time multi-view deconvolution
Source: Bioinformatics. 2015 Jun 25;31(20):3398–400. doi: 10.1093/bioinformatics/btv387 (PMC4595906; doi:10.1093/bioinformatics/btv387)
Supplement: Supplementary Data [file supp_btv387_SupplementaryText.pdf]

Supplementary material
Real-time multi-view deconvolution

Benjamin Schmid [1] and Jan Huisken [1,]

SUPPLEMENTARY NOTE 1: MEMORY REQUIREMENTS FOR MULTI-VIEW DECONVOLUTION

The multi-view Richardson-Lucy deconvolution algorithm iteratively updates the current estimate of the true image by the following formula:

$$\psi^{t+1} = \psi^t \prod_{v \in V} \frac{\phi_v}{\psi^t * P_v} * P_v^*$$

Assuming the two convolutions are performed in Fourier space, the memory required per pixel (of the isotropic fused dataset) is $22 * v + 12$ bytes, where v is the number of views:

description	data type	memory requirement (bytes/pixel)
kernel spectrum FFT(P_v)	complex	8 v
inverted kernel spectrum FFT(P_v^*)	complex	8 v
data ϕ_v	uint16	2 v
weights	float	4 v
estimate ψ	float	4
estimate spectrum FFT(ψ)	float	4
temporary buffer	float	4
		22 v + 12

SUPPLEMENTARY NOTE 2: IMPLEMENTATION

1. Summary of optimization methods

Preibisch *et al.* (2014) derived a number of optimizations to make traditional Richardson-Lucy multi-view deconvolution converge within less iterations. The implemented variants all used the following formula, but replaced X with the expressions given below:

$$\psi^{t+1} = \psi^t \prod_{v \in V} \frac{\phi_v}{\psi^t * P_v} * X$$

- Independent:

$$X = P_v^*$$

- Efficient Bayesian:

$$X = P_v^* \prod_{w \in W_v} P_v^* * P_w * P_w^*$$

- Optimization I:

$$X = P_v^* \prod_{v \in W_v} P_v^* * P_w$$

- Optimization II:

$$X = \prod_{v,w \in W_v} P_v^*$$

where ψ^t is the estimate at iteration t, ϕ_v is the observed data of view v, P_v is the PSF of view v, P_v^* is the flipped PSF of view v and W_v is the set of all virtual distributions of view v (see Preibisch *et al.* (2014) for more details).

2. Convergence and number of iterations

The optimizations derived in Preibisch *et al.* (2014) and listed above reduce the number of iterations the algorithm requires to converge. Convergence behavior of the different optimization variants were extensively studied in Preibisch *et al.* (2014) and apply likewise to our implementation. In practice, choosing the number of iterations is a trade-off between achieved quality and computation time. We therefore leave it to the user, who needs to make this decision based on the particular situation (e.g. if deconvolution is performed in real-time, a reduced number of iterations might be preferred for an increase in overall acquisition speed). To facilitate the decision, we provide a tool for interactively investigating different numbers of iterations on a single cross-section (see also the Fiji plugin manual).

3. CUDA workflow for plane-wise multi-view deconvolution

Our plane-wise multi-view deconvolution implementation uses multiple CUDA streams to overlap GPU computations with data transfer, such that not only copies to and from the GPU, but also loading and saving data from and to hard-drive come without additional cost. The implemented workflow is outlined below. Here, all processing and CUDA calls are asynchronous, i.e. non-blocking. Synchronization is achieved by calls to *cudaStreamSynchronize()*.

Algorithm 1: Workflow to interleave disk I/O and memory transfers with data processing.

nStreams = 3;
for $z \leftarrow 0$ **to** $nStreams$ **do**
  Initialize $streams[z]$;
  Load plane z from hard-drive into main memory;
end
for $z \leftarrow 0$ **to** $nStreams$ **do**
  $stream = streams[z \mod nStreams]$;
  if $z >= nStreams$ **then**
    $cudaStreamSynchronize(stream)$;
    Save deconvolved plane $(z - nStreams)$ to hard-drive;
    Load plane z from hard-drive into main-memory;
  end
  Copy plane z from main memory to GPU;
  for $it \leftarrow 0$ **to** $nIterations$ **do**
    Calculate Richardson-Lucy step on $stream$;
  end
  Copy plane z from GPU to main memory;
end
for $z \leftarrow (nPlanes - nStreams + 1)$ **to** $nPlanes$ **do**
  $stream = streams[z \mod nStreams]$;
  $cudaStreamSynchronize(stream)$;
  Save deconvolved plane z to hard-drive;
end

4. Libraries and dependencies

To efficiently calculate the Richardson-Lucy iteration step, convolutions were replaced by multiplications in Fourier domain. Fourier transformations were computed using the cuFFT library (https://developer. nvidia.com/cuFFT). Other arithmetic operations were implemented as custom CUDA kernel functions.

The entire workflow was implemented in the C programming language, using the CUDA specific extensions. The Fiji plugin was implemented in the Java programming language (Oracle Corporation). The C program was interfaced from Java using JNI (Java Native Interface).

The deployed plugin contains for each platform the corresponding binary library, which is statically linked agains the CUDA SDK. Additionally, the cuFFT library is bundled, which is required as a shared library.

Requirements for execution are a Nvidia graphics card that supports CUDA.

SUPPLEMENTARY TABLE 1: EXECUTION SPEEDS USING DIFFERENT GRAPHICS CARDS.

Graphics card	Execution time (s)		
	512^3 pixel	1024^3 pixel	2048^3 pixel
Quadro K2000	12.0	83.7	683.8
Tesla C2075	6.6	48.2	378.7
GeForce GTX 680	7.8	29.8	238.5
Tesla K40c	3.9	19.4	153.6
GeForce Titan black	4.0	21.1	152.3

Table 1. Comparison of execution speed for plane-wise deconvolution using different graphics cards. 10 iterations have been performed deconvolving two views. Processing was performed on a Dell T6100 workstation (Intel E5-2630 @2.3 GHz 2 processors, 64 GB RAM). Data sizes correspond to the sizes padded for Fourier convolution, i.e. the sum of the actual data size and the size of PSF.

SUPPLEMENTARY FIGURE 1: DECONVOLUTION RESULTS VIEWED ALONG THE DETECTION AXIS

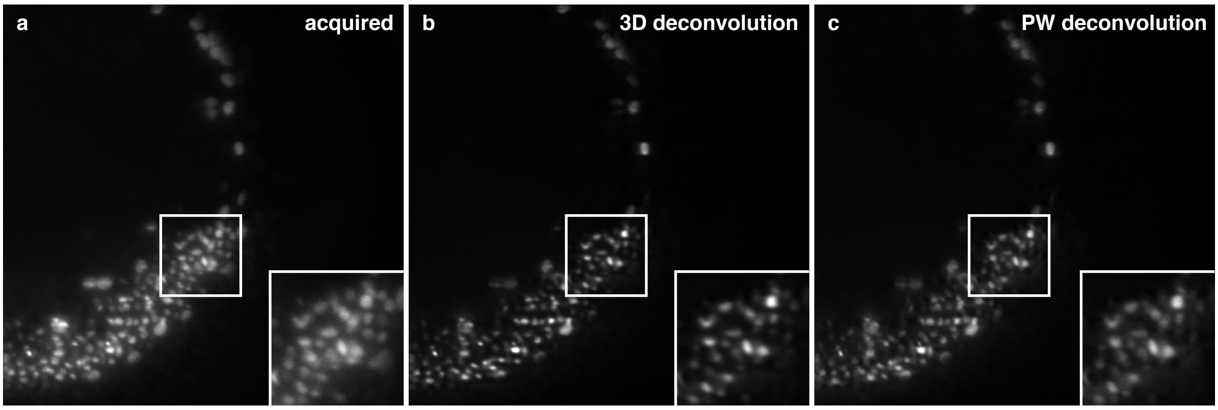

Supplementary Figure 1. Deconvolution results viewed along the detection axis. **(a)** Acquired data of the first view of a 9 hours post fertilization old *Tg(h2afva:h2afva-mCherry)* zebrafish embryo. **(b,c)** Multi-view deconvolution, performed **(b)** in full 3D and **(c)** plane-wise.

SUPPLEMENTARY FIGURE 2: VISUAL COMPARISON OF FUSION METHODS ON SIMULATED DATA

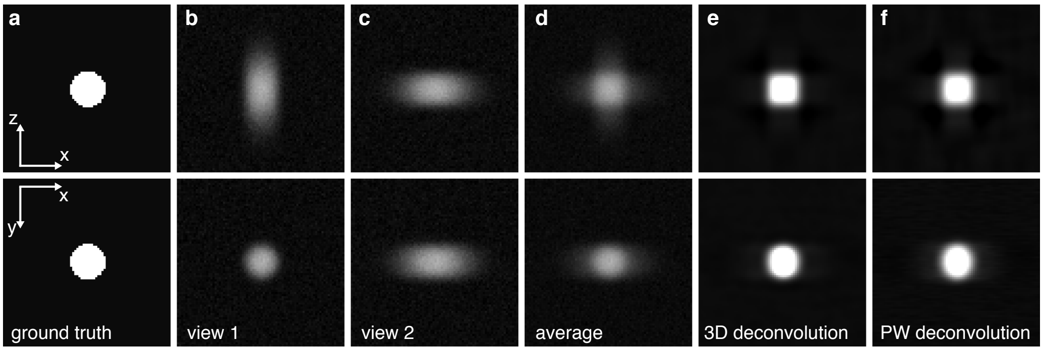

Supplementary Figure 2. Comparison of various fusion methods using a simulated data set. **(a)** Original data resembling a single nucleus of a *Tg(h2afva:h2afva-mCherry)* zebrafish embryo. **(b, c)** Simulated view 1 and view 2, generated by convolving the original data with an elongated PSF in z- and x-direction. Fusion by **(d)** averaging, **(e)** 3D multi-view deconvolution and **(f)** plane-wise multi-view deconvolution.

SUPPLEMENTARY TABLE 2: QUANTITATIVE COMPARISON OF FUSION METHODS ON SIMULATED DATA

	PW		3D	
Iteration	MSE	PSNR (dB)	MSE	PSNR (dB)
3	195.91	73.41	191.91	73.50
4	180.75	73.76	181.17	73.75
5	173.57	73.93	175.29	73.89
6	168.92	74.05	171.04	74.00
7	165.38	74.14	167.49	74.09
8	162.44	74.22	164.45	74.17
9	159.92	74.29	161.75	74.24
10	157.73	74.35	159.34	74.31

Table 2. Mean squared error (MSE) and peak signal-to-noise ratio (PSNR) of the reconstructed artificial data shown in supplementary figure 2 for different number of iterations. For all iterations, there is no significant difference between full 3D deconvolution and our plane-wise implementation. For comparison, the MSE for view 1 is 438.40 (PSNR = 69.91 dB), for view 2 448.36 (PSNR = 69.81 dB).

SUPPLEMENTARY FIGURE 3: COMPARISON OF DECONVOLUTION RESULTS ASSUMING A TILTED ROTATION AXIS

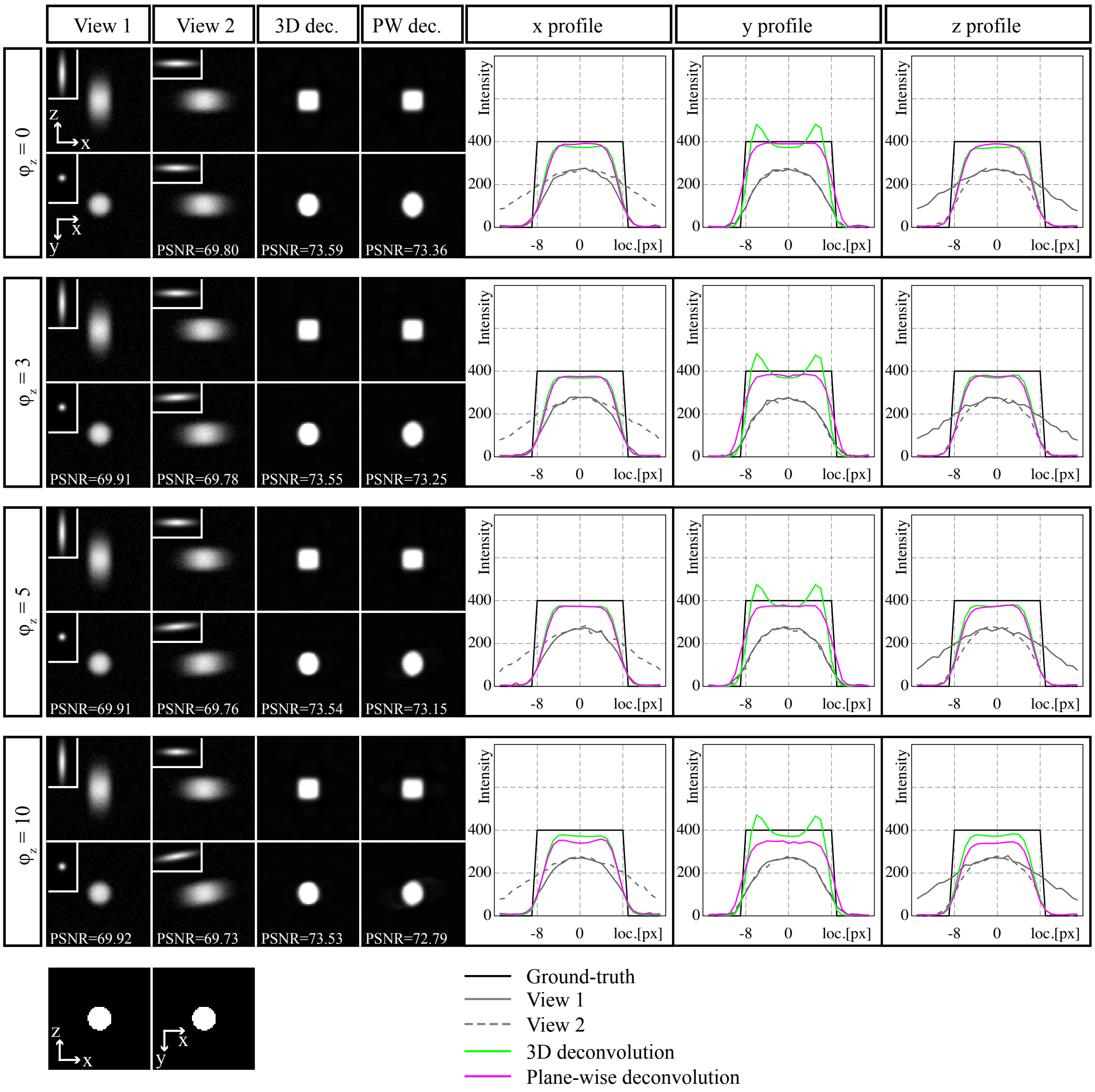

Supplementary Figure 3. Comparison of deconvolution assuming a tilted rotation axis. Simulated data were created as in Supplementary Fig. 2, but the rotation axis was tilted against the x/y plane by a number of angles. For each value, both views and the deconvolution results from the 3D deconvolution and our plane-wise implementation are shown, from top (top row) and along the detection axis of view 1 (bottom row). Peak signal-to-noise ratios (PSNR) are given for both methods (in dB). Line profiles are shown of the ground truth, the simulated data and the deconvolution results in all three dimensions. Even if the rotation axis is tilted by 10 degrees, 3D deconvolution is well approximated by our plane-wise implementation. On our microscope, the rotation axis is usually tilted by less than 1 degree.

SUPPLEMENTARY FIGURE 4: COMPARISON OF DECONVOLUTION RESULTS UNDER VARIOUS NOISE LEVELS

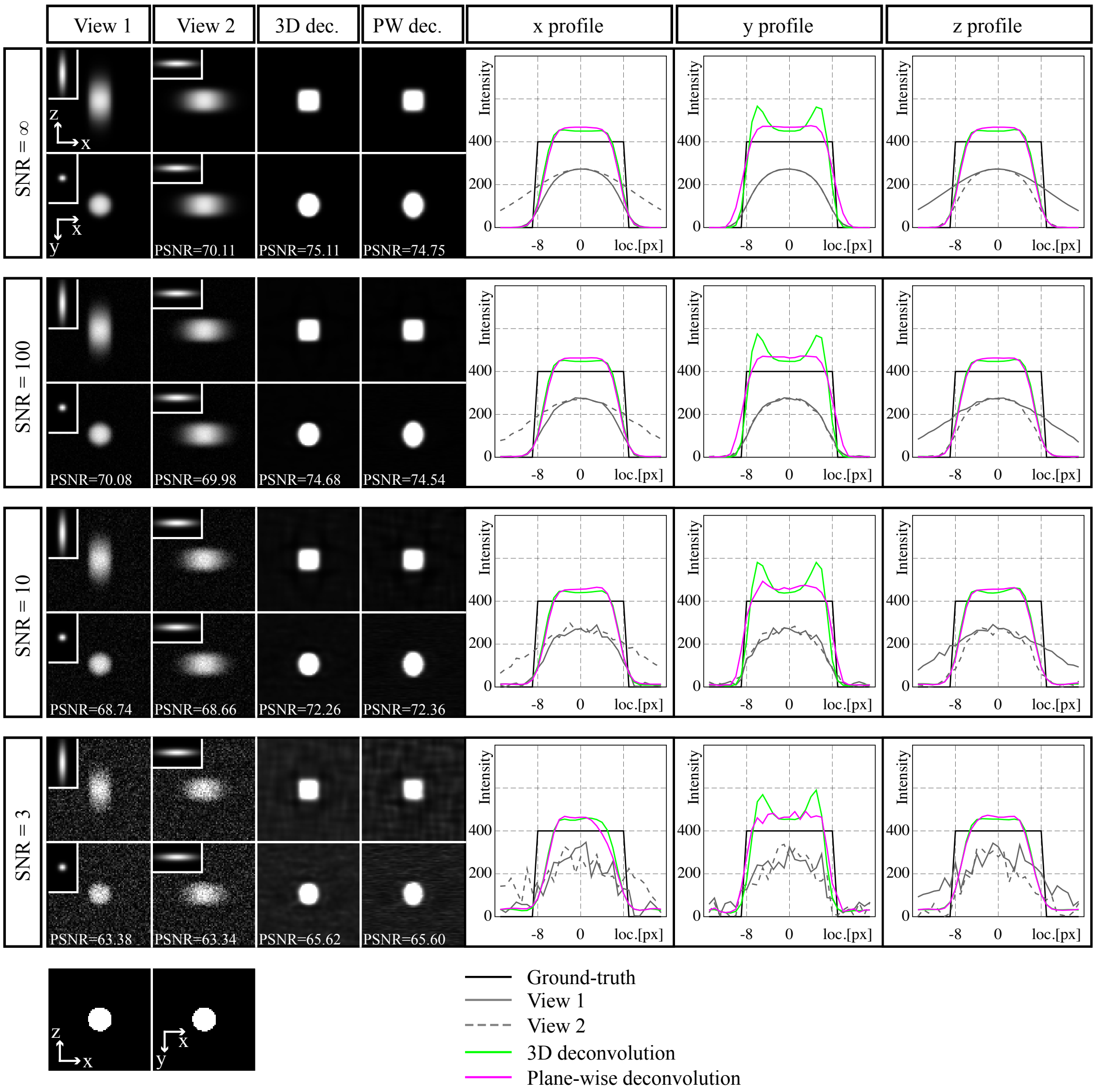

Supplementary Figure 4. Comparison of deconvolution results under various noise levels. Different amounts of Gaussian noise were added to the simulated data from Supplementary Fig. 2. For each signal-to-noise ratio (SNR), both views and the deconvolution results from the 3D deconvolution and our plane-wise implementation are shown, along the rotation axis (top row) and along the detection axis of view 1 (bottom row). Peak signal-to-noise ratios (PSNR) are given for both methods (in dB). For each SNR value, line profiles are shown of the ground truth, the simulated data and the deconvolution results in all three dimensions. Throughout all tested SNR values, results of plane-wise deconvolution closely resemble the results of the original 3D implementation.

SUPPLEMENTARY FIGURE 5: COMPARISON OF DECONVOLUTION RESULTS USING DIFFERENT PSFS

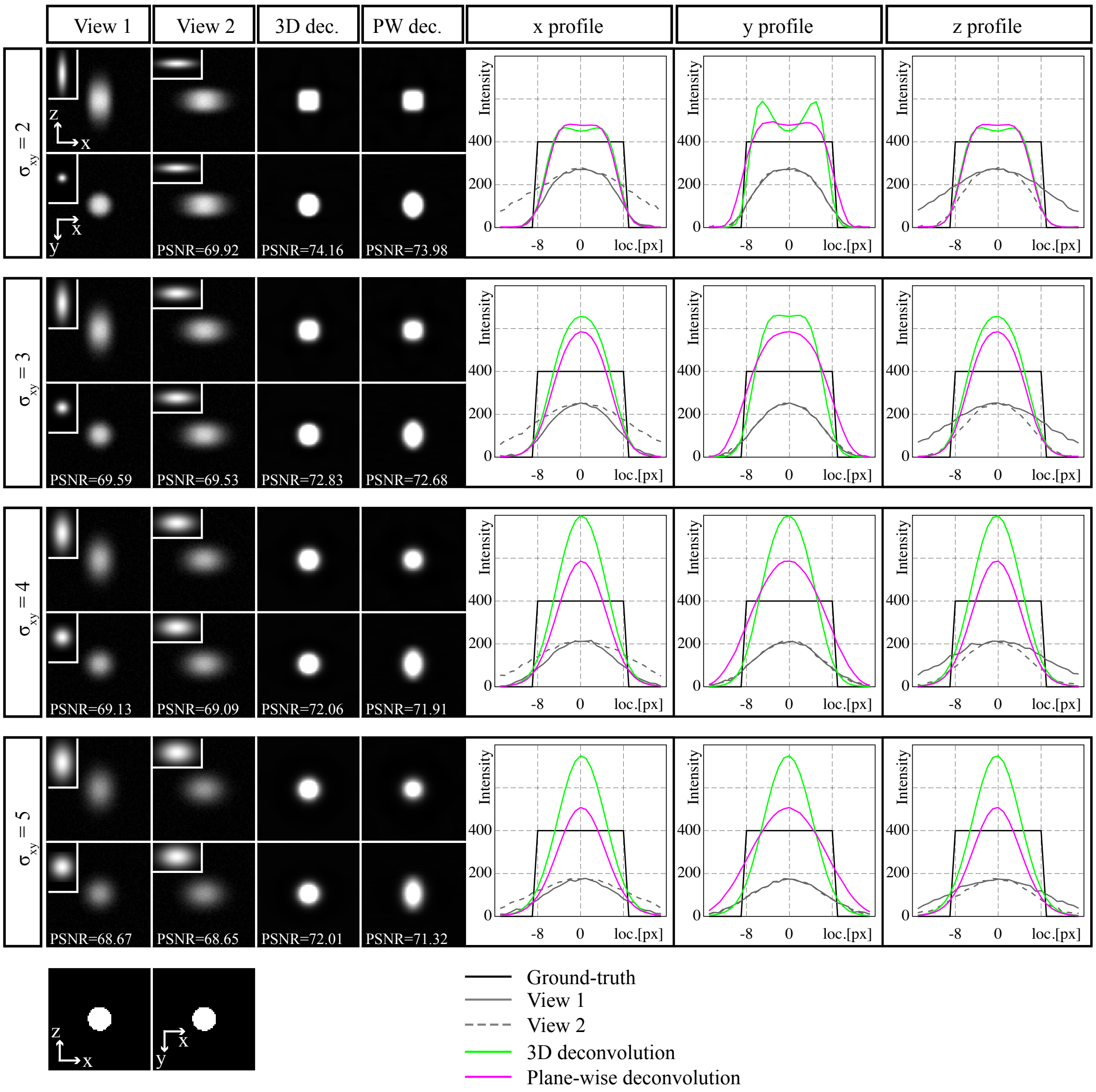

Supplementary Figure 5. Comparison of deconvolution results using different PSFs. Simulated data were created as in Supplementary Fig. 2, using Gaussian PSFs with a fixed axial standard deviation σ_z of eight pixels, as determined empirically on our microscope. Different values were used for the lateral standard deviation σ_{xy}. For each value, both views and the deconvolution results from the 3D deconvolution and our plane-wise implementation are shown, along the rotation axis (top row) and along the detection axis of view 1 (bottom row). Peak signal-to-noise ratios (PSNR) are given for both methods (in dB). Line profiles are shown of the ground truth, the simulated data and the deconvolution results in all three dimensions. While the results obtained by plane-wise and original 3D deconvolution are similar for small values of σ_{xy} below a value of two, they start to diverge for higher values. σ_{xy} on our microscopes was typically between 1.5 and 1.8 pixels.

REFERENCES

Preibisch, S., Amat, F., Stamataki, E., Sarov, M., Singer, R.H., Myers, E. & Tomancak, P. Efficient Bayesian-based multiview deconvolution. *Nat Meth* **11**, 645-648 (2014).
